# A Method of Controlling Preventable Diseases in Rural Districts*An address before the State Association of County and City Health Officers of Texas, at Austin, Texas, September 29, 1916.

**Published:** 1917-04

**Authors:** A. Caswell Ellis

**Affiliations:** Professor of the Philosophy of Education in the University of Texas


### TEXAS MEDICAL JOURNAL

DR. F. E. DANIEL,
Founder.

MRS. F. E. DANIEL,
Publisher and Managing Editor.

PUBLISHED MONTHLY.
Subscription:
$1.00 a Year.

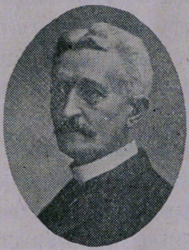

Established July, 1885.

VOL. XXXII No. 10
AUSTIN,
APRIL, 1917.

The publisher is not responsible for views of contributors.

*"All of good the past hath had,
Remains to make our own time glad."*—WHITTIER.

### Original Articles.

### A Method of Controlling Preventable Diseases in Rural Districts.*

BY DR. A. CASWELL ELLIS, PROFESSOR OF THE PHILOSOPHY OF EDUCATION IN THE UNIVERSITY OF TEXAS.

The only justification that I can find for a mere teacher appearing before this group of distinguished medical men and women is found in the fact that health campaigns are fundamentally campaigns of education, and therefore to be successful must take into consideration the principles of pedagogy, as well as those of sanitation and preventive medicine. In this paper I shall merely describe the methods that we have recently employed in East Texas and report the most significant results. This is not done under the impression that our methods are perfect or results ideal, but with the hope that you may offer suggestions that may help us to improve the method and to find a means of financing the work through its experimental stage until conservative governmental agencies will take it up on a scale commensurate with its importance.

Last spring the Texas State Health Department, the University of Texas Extension Department and the International Health Board agreed to co-operate with several East Texas counties in intensive rural health work, laying particular stress upon malaria, typhoid, hookworms and dysentery. These diseases were chosen for three reasons: First, their wide prevalence in those rural dis-

*An address before the State Association of County and City Health Officers of Texas, at Austin, Texas, September 29, 1916.

tricts; second, their causes are known and means of prevention are well established, simple, and not expensive; third, the same measures that protect against one of these diseases protect also against some of the others. The screening for malaria also keeps out the flies that bring typhoid and dysentery, the sanitary privy that protects from hookworm protects likewise against the spread of these two intestinal diseases through contaminated wells or through flies.

The campaign was opened by visiting the county medical association and through addresses, securing its endorsement of the plan. With a committee of physicians appointed by the county medical association our representatives presented the proposed plan of work to the county commissioners court. We proposed that the county appropriate $800 on condition the International Health Board furnish a medical director for the work and the board and the University Extension Department each furnish $800 toward defraying the expenses of the work. In four out of seven counties thus far the proposition has been accepted. All were interested.

This joint fund of $2400 pays for four months work simultaneously in three districts, each covering about 36 square miles. Having secured the funds and the co-operation of the local physicians, the next problem is to select the districts in which to work. Usually ten or a dozen places are visited, mass meetings held, and the plan of work explained. It is stated that the three districts that most nearly unanimously invite us to come to them will be the three chosen for the work. At each place a local committee of four is appointed, who carry around a sort of half-invitation and half-contract, and secure the signatures of as many heads of families as possible. This document invites us to take up the work in that community, offers free use of a small room for a laboratory, and pledges that the signers will allow us to inspect their homes, examine their families for hookworms and malaria, and that they will endeavor to carry out in good faith such directions and suggestions as our medical director offers. Knowing the strong disinclination of the individualistic, rural Texan to allow anyone to invade and criticise his home, and his equally strong dislike for following orders from any human being, I was astonished to find that in Leon county, where our first completed work was done, in four communities the head of every family signed the contract, and in several others the signatures of over 90 per cent of the families were secured. A few of these families failed to keep their pledges, but a surprisingly large percentage kept them, and none offered active opposition.

The three communities most receptive being selected, a small laboratory was fitted up in each, and a microscopist and carpenter left with the laboratory. The medical director passed periodically from one district to the other, giving his entire time to the three districts. Each laboratory contained a microscope and the necessary equipment for handling malaria and hookworm specimens, packages of medicine, and a few health charts, slides, and specimens dealing with the diseases emphasized.

With the aid of the local committee, the medical director and microscopist prepare a map showing the location of every road, creek, river, and home in the district, extending three or four miles in each direction from the central schoolhouse. The schoolhouse is used all through the campaign as the place for lectures and other meetings. With the map prepared, the real work begins.

The microscopist, with aid in special cases from the director, visits every home and while there does five things:

1. In conversation with the parents, he gathers the health history of each member of the family for the past year. Record is made not only of the number and variety of preventable diseases from which each member of the family suffered, but of the cost of doctor's bill and medicine, and of the money value of the time lost while in bed or too sick to work. Some of these family records will be given later.

2. The premises are inspected and suggestions offered as to what features are insanitary and how these can be remedied. The house may have no screens, or the screen doors may have no cloth flap to cover the cracks at the bottom and top, or the chimney may not be screened, or the wire may be twelve or fourteen mesh, which is only partially effective against anopheles. The relation of the mosquito to malaria, the value of screens and drainage and the use of quinine as a prophylactic measure are all discussed. The necessity for oiling the cistern and rain barrels, burying old tin cans, draining puddles around the well and the yard is also pointed out. The privy is examined, its insanitary features and the relation of these to hookworms, typhoid and dysentery are pointed out, and the best way to make the privy sanitary is indicated.

3. Five brief, non-technical, simply written and well-illustrated circulars and bulletins are left with the family, with the suggestion that they be studied. These deal with the cause and prevention of malaria, hookworms, and typhoid, with methods of screening, the proper use of quinine as prophylaxis and cure, and the construction of sanitary privies. The bulletins merely reinforce and enlarge what the visitor has said.

4. Blood smears are made from each member of the family and containers left with instructions to collect samples of the feces of each individual. These last are gathered later.

5. When it is desired, an appointment is made for the carpenter who is sent to assist in getting the screens and privy properly made.

After the visits, the microscopists and director examine all specimens under the microscope. After the blood and feces have been examined, a formal report is sent by the medical director to the head of the family, stating which members of the family are infected. Those with hookworms are advised to report to the laboratory for treatment, and those with malaria are advised to consult their family physician and to continue their treatment until the parasite is eradicated. In order to secure a desirable uniformity in treating the malaria, the medical director meets with the local doctors and through discussion tactfully gets all to agree to follow the plan of prescribing the use of 10 grains of quinine a day for thirty days after the chill and fever are broken, as this seems the most certain way to eradicate the parasites from the internal organs. These who follow treatment are re-examined after four to eight weeks and results again communicated.

Those with hookworms are given chenopodium free and are reexamined and, if necessary, treated weekly until cured.

All the while, night lectures, illustrated by charts, lantern slides, and specimens are given each two weeks at the schoolhouse by the medical director, or the Director of Extension, or both. Separate lectures are given to whites and negroes. These lectures deal with general sanitation, diet, tuberculosis, malaria, hookworms, typhoid, dysentery, and any disease that may be of local interest, such as pellagra, for example. The large numbers that attend these lectures are most gratifying. I gave four of these lectures myself and had full houses every time, except once when the meeting unwisely was set so as to conflict with both a local revival and lodge night. In one case I remember that every seat was taken, and many were standing at the back and even out of doors where they could see and hear through the open windows.

So much for the method. Now the question remains, what were the results of this "spring drive" against disease with big guns, machine guns, Zeppelins, submarines, bayonets, liquid fire, and poisonous gases—bulletins, lectures, personal persuasion, demonstration of cures, screens, drainage, quinine, prophylaxis, etc.?

First, the lasting value of the preliminary hookworm work done by the Rockefeller Commission several years ago is made plain. In districts numbers 1 and 2, in which hookworm work was done several years ago, 7.8 per cent of the population were found infected, whereas in district number 3, in which no work had been done, 25 per cent were found infected.

Second, it has been shown that in four months' time the hookworm infection can be reduced from 25 per cent to 3 per cent by voluntary effort, and that a community found with 103 homes, none having sanitary privies, only nineteen having insanitary ones, and eighty-four having no privy at all, may be induced to build 101 sanitary privies without any process of law.

Third, it has been shown that 75 per cent of the families in these malarial districts that had properly screened homes entirely escaped malaria, and 58 per cent of those with inadequate screens escaped, whereas only 5 per cent of those with no screens escaped.

Fourth, it is shown that it is much more difficult to induce the rural population to add screens than it is to persuade them to build sanitary privies. District number 3, for example, that built and remodeled 101 privies, put screens on only 10 homes. There is reason to believe that the number of screens will be gradually increased later. Many tenant homes are so poorly constructed that it is well-nigh impossible to screen them. Texas ought to have a building law requiring that every tenant house should have a sanitary privy, a sanitary water supply, and a dwelling at least capable of being screened.

Fifth, the value of the proper use of quinine in treating malaria and the inefficiency of the usual use of it is again clearly shown. Of those with malarial infection of long standing, who claimed to follow our directions 84 per cent were cured, while of those who took the quinine according to their own notions only 16 per cent were cured.

Sixth, there is a clear demonstration of the harmlessness of quinine, and practicaly perfect protection against malaria afforded by quinine when taken as a prophylactic, either five grains every day or ten grains per day on two successive days each week. Its use was scorned by a large per cent of the population until they saw families who used it as directed remaining perfectly well, while they themselves were constantly down with malaria. I have here, for example, the family records of four families with a total of twenty-eight persons, every one of whom had malaria last year and all of whom together had a total of 308 different attacks, each attack involving one or more chills or fevers. They are living in the same homes this year, but are taking preventive doses of quinine, and not one of them, at the last report, had suffered an attack this year. Not a single one of our working force had an attack as long as the quinine was taken. Two of them neglected it at the last, and one of those went down with a violent attack within ten days of the time he omitted the quinine, and the other within about thirty days.

Finally, we have the most nearly accurate account yet secured of the actual kinds and amounts of preventable diseases present in our lowland, coastal plain country. In the approximately 200 white families studied, there were four deaths from malaria last year, and an average of 2.4 attacks per person. There was a money loss from preventable disease of $77 per family, on account of doctors' bills, medicine, nursing, and loss of time from work. There was a per capita loss of $15.71 for the whites. The negroes had just as much sickness, but because of neglect the cost was about half that of the whites. The total immediate cost of preventable disease, as far as this could be recalled at the end of the year by 179 white and 119 negro families studied, was $17,703. Of this, malaria caused the loss of $12,258. As there are about a million people living along the coastal plain of Texas, equally exposed to these attacks, it means that last year one-fourth of the population of Texas alone paid a preventable disease tax of about fifteen million dollars, including a malaria tax of about ten million. In addition, they suffered the pain of more than two million attacks of malaria and lost over four thousand loved ones by death. East Texas is no worse nor better than the rest of the Gulf and Atlantic coastal plain, at least as far up as Virginia, and the valleys of the Mississippi, Missouri, Ohio, and a thousand and one other rivers and creeks in the temperate and semi-tropical parts of America. Multiply the millions of cases of suffering and the millions of money loss that are now proven in Texas by the number of millions inhabiting the anopheles infested coastal plain and river and creek botoms, and you will have some idea of the hundreds of millions of money and the frightful tribute of human pain and misery that we stupidly pay each year, when our experience seems to show that for less than one-seventh of the actual amount lost each year we can immediately and permanently prevent more than half of this loss, and in a few years practically all of it.

The National government spends hundreds of thousands per year on hog culture and hog cholera, hundreds more on cattle culture and cattle ticks, and yet the fund allowed Dr. Von Ezdorf this year for malaria work is just nineteen thousand dollars for this vast empire. When I plead with Congress for a small sum to start this work in Texas, I was solemnly assured by a distinguished member that it might prove a very bad precedent. That if the work succeeded, the whole country would want such help, and that it would cost a billion dollars before the country was freed of these diseases. The fact that that billion would save fifty thousand lives and a billion dollars per year made no dent in the crust of his mind. The same government that refused to vote $50,000 for rural sanitation voted an army and navy program of over six hundred million; and, when a dozen citizens were murdered by a band of irresponsible bandits, promptly shipped 50,000 trained soldiers to South Texas at a cost of over two million dollars, and a little later, when a dozen or so soldiers were shot sent 100,000 more soldiers at an expense of about ten million dollars, and has kept the entire number there month after month ever since, at a cost of five or ten million per month—and all that was done in South Texas while 4000 citizens were being murdered in East Texas by easily controlled plant and animal parasites, without the government making any reasonable effort to save them. That may be the esablishetd precedent, but it is certainly a besottedly stupid precedent.

This important health work could never have been begun except for the liberal appropriations wisely granted the University and the State Health Department by the last Legislature and approved by Governor Ferguson. For years our State has stupidly neglected to protect the health of her people and has lost hundreds of millions of dollars and tens of thousands of lives by this penny-wise and pound-foolish parsimony—it is not economy. Even now, some are criticising our Governor because of the increased taxes necessary to protect and educate our citizens and build up the civilization of this great State. Let us put a stop to such demagogy and as sensible men and women recognize that it will cost something to have the blessings of health, education, and civilization, but that it costs far more to do without them. Let us give our aid to that wise policy of Governor Ferguson which has made possible the beginning in this health work and similar beginnings in other lines of education and protection.

You medical men who have it in your power to make it so that no public man will dare to place dollars above human life, or reducing the tax rate above educating the people, I beg of you to use your influence with your neighbors and your legislators to support your State Health Department and your State University in this important health work, which has only just begun. The National government, the State, the county, and we ourselves have each a duty to perform in this matter, and, if we perform that duty, Texas and the entire country can in ten years time be as free of malaria, typhoid, hookworms, and dysentery as we are now free of cholera and bubonic plague.

TABLE I.—HEALTH RECORD, MAY 1, 1915 TO MAY 1, 1916.

| Community. | Malaria. | | | | | | | | | | Other Preventable Diseases. | | | | |
|---|---|---|---|---|---|---|---|---|---|---|---|---|---|---|---|
| | Number of families. | Number of persons. | Number sick. | Number spells of Malaria. | Days sick in bed. | Additional days too sick to work. | Total days lost. | Doctors' bills and medicine. | Cost of time of workers lost. | Died, 1915. | Number sick. | Days sick in bed. | Days too sick to work. | Doctors' bills and medicine. | Cost of time of workers lost. |
| Whites— | | | | | | | | | | | | | | | |
| Vanitia | 53 | 273 | 224 | 1,485 | 1,857 | 524 | 2,381 | $ 700 | $ 1,796 | 1 | 11 | 149 | 38 | $ 38 | $ 117 |
| Leona | 36 | 183 | 53 | 189 | 303 | 189 | 492 | 148 | 407 | 1 | 42 | 442 | 846 | 458 | 782 |
| Marquez | 90 | 418 | 160 | 468 | 1,565 | 1,832 | 3,397 | 1,889 | 3,801 | 2 | 33 | 746 | 1,316 | 2,397 | 1,127 |
| Total | 179 | 874 | 437 | 2,142 | 3,725 | 2,545 | 6,270 | $ 2,807 | $ 6,004 | 4 | 86 | 1,337 | 2,200 | $ 2,893 | $ 2,026 |
| | | | | | | | | $8, | 811 | | | | | $4, | 919 |
| Negroes— | | | | | | | | | | | | | | | |
| Vanitia | 29 | 138 | 101 | 424 | 650 | 908 | 1,558 | $ 267 | $ 211 | 0 | 9 | 64 | 89 | $ 36 | $ 27 |
| Leona | 57 | 313 | 194 | 605 | 1,605 | 776 | 2,381 | 403 | 1,299 | 0 | 41 | 223 | 409 | 97 | 337 |
| Marquez | 33 | 129 | 70 | 151 | 537 | 539 | 1,076 | 312 | 955 | 0 | 1 | 2 | 14 | 4 | 25 |
| Total | 119 | 580 | 365 | 1,180 | 2,792 | 2,223 | 5,015 | $ 982 | $ 2,465 | 0 | 51 | 289 | 512 | $ 137 | $ 389 |
| | | | | | | | | $3, | 447 | | | | | $5 | 26 |
| Grand total | 298 | 1,454 | 802 | 3,322 | 6,517 | 4,768 | 11,285 | $ 3,789 | $ 8,649 | 4 | 137 | 1,626 | 2,712 | $ 3,030 | $ 2,415 |
| | | | | | | | | $12,258 | | | | | | $5,445 | |

| | |
|---|---|
| Total cost of preventable sickness of 179 white families (874 persons) | $13,730 00 |
| Cost of preventable sickness per family (doctor and medicine, $32 00; loss of labor, $45 00) | 77 00 |
| Cost of preventable sickness per white inhabitant | 15 71 |
| Total of preventable sickness of 119 negro families (580 persons) | 3,975 00 |
| Cost of preventable sickness per family (doctor and medicine, $9 40; loss of labor, $24 00) | 33 40 |
| Cost of preventable sickness per negro inhabitant | 6 90 |

**TABLE II.**

**Report on Work for the Relief and Control of Hookworms.**

| Community. | Census. | Given first examination. | Refused examination. | Removed before examination. | Infected on first examination. | Given first treatment. | Cured. | Treatment resumed. | Removed before cured. |
|---|---|---|---|---|---|---|---|---|---|
| Whites— | | | | | | | | | |
| District No. 1 | 619 | 571 | 44 | 4 | 62 | 61 | 56 | 4 | ........ |
| District No. 2 | 716 | 669 | 28 | 2 | 35 | 34 | 32 | ........ | 1 |
| District No. 3 | 494 | 451 | 43 | ........ | 113 | 113 | 98 | ........ | ........ |
| Negroes— | | | | | | | | | |
| District No. 1 | 141 | 76 | *23 | 70 | 12 | ........ | ........ | ........ | ........ |
| District No. 2 | 140 | 49 | 57 | 31 | 12 | ........ | ........ | ........ | ........ |
| District No. 3 | 103 | 19 | †82 | 19 | 12 | ........ | ........ | ........ | ........ |

*(Inclusive of 5 extra closets.) †(Inclusive of 8 extra closets.)

**TABLE III.**

**Health Survey, Leon County, Texas, 1916—Malaria—Screening of Residences at Beginning of Survey, April, 1916.**

| Community. | Number of homes. | Number of houses well screened. | Number of homes improperly screened. | Number of homes without screens. | Number homes giving history of Malaria, May 1, 1915–1916. | | | Number of homes giving no history of Malaria. |
|---|---|---|---|---|---|---|---|---|
| | | | | | Well screened. | Improperly screened. | Without screens. | |
| District No. 2 | 140 | 3 | 22 | 115 | 1 | 9 | 103 | 27 |
| District No. 1 | 141 | 1 | 29 | 111 | 0 | 10 | 111 | 20 |
| District No. 3 | 102 | 0 | 8 | 94 | 0 | 6 | 90 | 6 |
| Totals..... | 363 | 4 | 59 | 320 | 1<br>25% | 25<br>42% | 304<br>95% | 53 |

Disinterested Advice.—Mr. Tyte-Phist (at the club)—"By the way, doc, what is good for indigestion?"

Doctor (fellow clubman): "Well, a Welsh rabbit is sometimes good for about three days of it."

---

Tightwad.—"I understand that Mr. Pinchpenny has been operated on for appendicitis," remarked Miss Cayenne.

"Yes. It's the first time any one was known to get anything out of him."

"And even then they had to give him chloroform to get that."

